# Specific Detection of Serum Antibodies against BKPyV, A Small DNA Tumour Virus, in Patients Affected by Choroidal Nevi

**DOI:** 10.3389/fmicb.2017.02059

**Published:** 2017-10-23

**Authors:** Silvia Pietrobon, Ilaria Bononi, Francesca Lotito, Paolo Perri, Sara Violanti, Elisa Mazzoni, Fernanda Martini, Mauro G. Tognon

**Affiliations:** ^1^Section of Pathology, Oncology and Experimental Biology, Department of Morphology, Surgery and Experimental Medicine, School of Medicine, University of Ferrara, Ferrara, Italy; ^2^Section of Ophthalmology, Department of Biomedical Sciences and Specialized Surgeries, School of Medicine, University of Ferrara, Ferrara, Italy

**Keywords:** choroidal nevus, serum, antibody, prevalence, titer, BKPyV

## Abstract

Ocular or choroidal nevus (CN) is a rare benign neoplastic lesion of the eye. The cause of CN onset/progression, which arises from the transformation of ocular melanocytes, is not known. A fraction of CN patients may develop uveal melanoma. The objective of this study was to investigate the association between CN and BK polyomavirus (BKPyV), a small DNA tumor virus. Serum IgG antibodies which react with BKPyV antigens were analyzed. An indirect E.L.I.S.A. using synthetic peptides that mimic BKPyV antigens was employed. Serum antibodies against BKPyV were also investigated by haemagglutination inhibition (HAI) assay. Sera were from CN patients and healthy subject (HS) were the control. A statistically significant higher prevalence of antibodies against BKPyV capsid protein antigens in serum samples from CN patients was detected, compared to HS, using two independent techniques, indirect E.L.I.S.A. and HAI (87.3% CN vs. 62.1% HS and 91.5% CN vs. 64.4% HS, respectively; *p* < 0.005). Our data suggest an association exists between CN and BKPyV indicating that this small DNA tumor virus could be responsible in the onset of this benign neoplastic lesion affecting eye melanocytes. This investigation reports the association between choroidal nevi and BKPyV infection for the first time. These data are innovative in this field and may represent a starting point for further investigation into the putative role of BKPyV in CN onset/progression.

## Introduction

Ocular or choroidal nevi (CN) are benign melanocytic lesions of the eye. In Europe, their prevalence is about 5% in Caucasians. Ocular nevus rarely evolves into overt melanoma of the eye. Indeed, only 0.01% (1/8,800; Qiu and Shields, 2015) of patients affected by CN develop uveal melanoma (UM).

The onset of this benign neoplastic lesion is caused by the transformation of melanocytes, which are cells of neural origin specialized in the production of the pigment melanin. During embryogenesis melanocytes migrate from the neural crest to other tissues in the host. In adults, they are present on the skin, stria vascularis of the ear, meninges and in the uveal component of the eye. The function of melanin, as a pigment, is to protect from sun light. Indeed, melanin acts as a shield to protect skin keratinocytes from ultraviolet (UV) rays. CN etiopathogenesis is not completely understood. As in other human neoplasia, a multistep process of cell transformation accounts for CN onset, with UV being the main physical mutagenic agent. However, other chemical, physical and biological transforming agents, such as viruses with oncogenic potential should be taken into consideration (Jovanovic et al., 2013).

BK Polyomavirus (BKPyV) is characterized by double-stranded circular DNA of about 5.15 Kb. BKPyV was isolated for the first time from the urine of a kidney transplant patient (Comar et al., 2004; Helanterä et al., 2016). BKPyV is now considered an ubiquitous virus with a world-wide infection rate range of 65–90%, depending on the study and population analyzed. BKPyV primary infection occurs early in life, during childhood. It subsequently remains lifelong in the host in a latent state. However, BKPyV may reactivate both in immune-compromised patients as well as in healthy subjects (HS; Becker et al., 2013). In kidney transplant patients, BKPyV may cause haemorrhagic cystitis and allograft rejection (Hashida et al., 1976; Mininberg et al., 1982).

BKPyV has transforming and oncogenic properties, which are due to the activities of two viral oncogenes, named large T antigen (Tag) and small t antigen (tag). In this context, it is worth recalling that Tag induces chromosomal aberrations and stimulates cellular gene expression. BKPyV transforms different animal and human cell types *in vitro*, whereas, it induces tumors of different histotypes in experimental animals. Due to its oncogenic potential, BKPyV is considered a small DNA tumor virus (Becker et al., 2013). Its footprints have been detected in human cancers, such as brain and bone tumors, insulinomas, Kaposi's sarcomas, carcinomas of the urinary, and genital tracts (Tognon et al., 2003). Moreover, in some studies, an association between human cancers and BKPyV was reported, due to high antibody titres against this polyomavirus being revealed in oncologic patients (Becker et al., 2013).

Thus far, detection of antibodies against BKPyV has been performed using serological methods, mainly employing virus-like particles (VLPs) or soluble recombinant VP1 as antigens. Data obtained using these immunological techniques have been hampered by some cross-reactivity among VPs from three different polyomaviruses, such as BKPyV, JCPyV, and SV40 (Carter et al., 2003; Viscidi et al., 2003; Barbanti-Brodano et al., 2004; Lundstig et al., 2005; Kjaerheim et al., 2007; Ribeiro et al., 2010; Corallini et al., 2012; Pietrobon et al., 2017). Indeed, VPs from these three polyomaviruses are similar in amino acid sequences, showing ~70% homology (Corallini et al., 2012; Pietrobon et al., 2017). This high homology is responsible for cross-reactivity among these three polyomaviruses, which in turn gave non-specific serologic data (Carter et al., 2003; Viscidi et al., 2003; Barbanti-Brodano et al., 2004, 2006; Lundstig et al., 2005; Kjaerheim et al., 2007; Kean et al., 2009; Ribeiro et al., 2010; Corallini et al., 2012; Taronna et al., 2013).

To circumvent the cross-reactivity among BKPyV, JCPyV, and SV40 we have developed an indirect E.L.I.S.A. with synthetic peptides mimicking specific and unique BKPyV VP1 antigens (Pietrobon et al., 2017). Our immunologic data indicate that this E.L.I.S.A. is specific to BKPyV, without cross-reactivity with JCPyV and SV40 (Pietrobon et al., 2017).

In this study, the association between CN and BKPyV was investigated. To this purpose serum antibodies against BKPyV were investigated using an indirect E.L.I.S.A., which employs two synthetic peptides of BKV VP1, as specific antigens (Pietrobon et al., 2017). To verify the data obtained using the indirect E.L.I.S.A., serum samples were also tested with the well-established method, known as Haemagglutination Inhibition (HAI) assay (Portolani et al., 1974; Corallini et al., 2012).

## Materials and methods

### Serum samples

Sera (*n* = 279) were collected from patients affected by choroidal nevi (CN; *n* = 71) and healthy subjects without ocular nevi (HS; *n* = 208), attending the Eye Clinic of the University Hospital of Ferrara, Italy. Serum samples were collected after routine analysis from discarded samples from the Clinical Laboratory Analysis before incineration.

The two cohorts included patients/individuals of both genders, with the same median age (range = 64–67 years), Table 1.

**Table 1:** Prevalence of serum IgG antibodies reacting with BKPyV VP1 mimotopes detected in CN and HS.

**Human serum**	**Number of subjects/patients**	**Median age ± DS**	**Male (%)**	**CN/HS ratio**	**Number of positive samples (%)**		
					**VP1-L**	**VP1-M**	**VP1 (L+M)**
CN	71	67 ± 11	38		66 (93.0)	63 (88.7)	62 (87.3)^***^
HS1	87	69 ± 13	41	1:1	56 (64.4)	65 (74.7)	54 (62.1)
HS2	144	66 ± 11	45	1:2	102 (70.8)	103 (71.5)	92 (63.9)
HS3	208	64 ± 10	44	1:3	151 (72.6)	152 (73.1)	135 (64.9)

Human sera were from patients affected by choroidal nevi (CN) and healthy subjects (HS). The prevalence of BKPyV antibodies in CN patients is statistically significant higher than that detected in HS1, HS2, and HS3(

*** *p < 0.001)*.

*Statistical analysis was performed using the χ^2^ test*.

Anonymously collected sera were coded with indications of age, gender, and pathology only. Written informed consent was obtained from the subjects after explanation of the nature and possible consequences of the study. All patients/subjects signed informed consent at the time of hospital admission. The research followed the tenets of the Declaration of Helsinki. The Ethics Committee, Ferrara, approved the study.

### Synthetic peptides

Computer assisted analyses enabled us to identify two specific BKPyV peptides, selected from the late viral region by comparing BKPyV VP1 with the corresponding amino acids from JCPyV and SV40, which are highly homologous to BKPyV, as well as with other, less homologous, polyomaviruses (web site, http://blast.ncbi.nlm.nih.gov; Pietrobon et al., 2017).

Experimental data indicate that the selected BKPyV VP1 peptides, employed as antigens in indirect E.L.I.S.A.s, do not cross-react with JCPyV and SV40 hyperimmune sera (Pietrobon et al., 2017).

The two BKPyV VP1 synthetic peptides are:

BKPyV VP1 L: NH2 – LKLSAENDFSSDSPERK–COOH
BKPyV VP1 M: NH2–MLNLHAGSQKVHEHGGGK–COOH

### Indirect enzyme-linked immunosorbent assay (E.L.I.S.A.)

Indirect E.L.I.S.A. was developed and standardized to detect specific antibodies against BKPyV in human sera using VP1 L and M synthetic peptides as mimotopes. A human peptide, represented by the neuropeptide S (hNPS), a.a sequence SFRNGVGTGMKKTSFQRAKS, which is BKPyV un-related, was used as a negative peptide in all indirect E.L.I.S.A. reactions (Corallini et al., 2012; Tognon et al., 2016). *Peptide coating*. Plates were coated with 5 μg of the selected peptide, for each well, diluted in 100 μL of Coating Buffer (Candor Bioscience, Wangen im Allgäu, Germany) at 4°C for 16 h. *Peptide blocking*. Blocking was made with 200 μL/well of the Blocking Solution (Candor Bioscience, Wangen im Allgäu, Germany) at 37°C for 90 min.

#### Primary antibody adding

Wells were covered with 100 μL of serum sample, diluted 1/20, in Low Cross-Buffer (Candor Bioscience, Wangen im Allgäu, Germany). The positive BKPyV control was represented by immune rabbit serum containing anti-BKPyV antibodies; negative controls were represented by immune sera anti-SV40 and anti-JCPyV and three human serum samples which were found to be BKPyV-negative in our previous investigation both by indirect E.L.I.S.A.s and Haemagglutination Inhibition Assay (H.I.A.; Pietrobon et al., 2017). Each sample was analyzed three times in replica experiments.

#### Secondary antibody adding

After three washing treatments, wells were covered with a solution (200 μL) containing a goat anti-human or anti-rabbit IgG heavy and light chain specific peroxidase-conjugate (Calbiochem-Merck, Darmstadt, Germany), diluted 1:10,000, in Low Cross-Buffer (Randhawa et al., 2008).

#### Dye treatment and spectrophotometric reading

Wells were treated with 100 μL of 2,2′-azino-bis 3-ethylbenzthiazoline-6-sulfonic acid (ABTS) solution (Sigma-Aldrich, Milan, Italy), for 45 min. at RT, and then plates were read on the spectrophotometer (Thermo Electron Corporation, model Multiskan EX, Helsinki, Finland) at a wavelength (λ) of 405 nm. The color intensity in wells where immunocomplexes were formed was determined by optical density (OD).

#### Cut-off determination

The cut-off point was determined in each assay using the mean value of OD reading for three negative controls, added to the standard deviation multiplied three times (+3 *SD*). Sera with antibodies against BKPyV were considered VP1-positive upon reacting to both peptides of the late region and when sera that had been analyzed three times by indirect E.L.I.S.A. testing gave the same positive result (Corallini et al., 2012; Mazzoni et al., 2012; Martini et al., 2013; Taronna et al., 2013).

### Viral working stock

BKPyV viral stock was obtained in infected VERO cells, as previously done (Corallini et al., 2012). Briefly, monolayers of VERO cells grown in T75 tissue culture vessels, with RPMI 1,640 medium with 5% fetal bovine serum (FBS) and 1% penicillin-streptomycin, 90% confluent, were infected at the multiplicity of infection (m.o.i.) of 10^−4^ plaque forming unit (P.F.U.)/cell. BKPyV-infected cells were collected 3 weeks after infection. The viral titre, determined by Haemagglutination (HA) assay, was 1.6 × 10^4^ haemagglutinating units (HAU), corresponding to 1.6 × 10^8^ P.F.U./ml (Corallini et al., 2012; Pietrobon et al., 2017).

BKPyV working stock was employed as viral antigens in both HA and HAI assays (Corallini et al., 2012).

### Haemagglutination (HA) assay

BKPyV titre was evaluated by HA with a solution of human erythrocytes, group 0, Rh+. These erythrocytes agglutinate in the presence of a specific concentration of BKPyV virions, i.e., they form a network that maintains red cells in suspension. In the absence of or with a low virion concentration, red cells precipitate and form a red spot on the bottom of the well (Mazzoni et al., 2012; Pietrobon et al., 2017).

Serial dilutions of the viral stock were carried out in plates, 96 round wells (Nunc, CelBio, Milan) in PBS 1x, from 1:10 at 1:5,120 dilution, in 100 μL of final volume. Then, 50 μL of 0.5 or 1% erythrocytes, were added to each viral dilution. Plates were incubated at +4°C and the H.A. titre was read 4 h later when the control, represented by erythrocytes in PBS only, had completely sedimented on the well bottom. The highest dilution of BKPyV, which gives the complete haemagglutination was considered to contain 1 HAU (Portolani et al., 1974; Corallini et al., 2012).

### Haemagglutination inhibition (HAI) assay

HAI tests the ability of a BKPyV immune serum to inhibit the haemagglutination capability of the virus. HAI employs serial dilutions (1:16, 1:32, 1:64, 1:128) of serum to determine the antibody titre. Sera were heated at 56°C for 30 min and treated with NaIO_4_ (0.1 M) to remove non-specific inhibitors. Specifically, 30 μL of sera was added to 15 μL of NaIO_4_ (1:2), in plates 96 round wells (Nunc, CelBio, Milan) and incubated at RT for 30 min. As a final step, 15 μL of 5% glycerine was added to each serum. Serial dilutions of the serum in PBS 1x, from 1:16 to 1:128, were mixed with 8 HAU of BKPyV as antigen. Mixtures were kept at RT for 1 h. Then, 2 volumes of 0.5% erythrocytes group 0+ were added, and plates were incubated at +4°C for 4 h. As a final step, the HI-antibody titre was determined, as described above (Portolani et al., 1974; Corallini et al., 2012).

### Statistical analysis

The prevalence of BKPyV-positive sera in choroidal nevi (CN) was compared with that in healthy subjects (HS). The statistical tests employed were (i) a one way t-student test to compare serologic profile (OD); (ii) Chi square testing was used to compare binary variables; (iii) Fisher's exact test was used to compare the prevalence of antibodies anti-BKPyV in different groups. The *P* < 0.05 value was considered statistically significant. All statistical analyses were performed using Prism software (GraphPad, San Diego, CA).

## Results

### Detection of BKPyV serum antibodies by indirect E.L.I.S.A. with specific BKPyV VP1 mimotopes in patients affected by choroidal nevi

Human sera, from CN patients (*n* = 71) and three groups of normal subjects, employed as controls indicated as HS1 (*n* = 87), HS2 (*n* = 144), and HS3 (*n* = 208; Table 1), were analyzed for IgG antibodies reacting to BKPyV VP1 mimotopes, known as VP1 L and VP1 M.

To this purpose, an indirect E.L.I.S.A. was employed using synthetic peptides as viral antigens (Pietrobon et al., 2017). These mimotopes correspond to specific BKPyV VP1 antigenic epitopes. As a control reaction, an unrelated BKPyV human synthetic peptide, known as hNPS, was used as a negative control (Corallini et al., 2012).

In the first step of this investigation, the prevalence of BKPyV antibodies was analyzed in 71 CN and 87 HS1 serum samples in a 1:1 ratio cases/controls.

A prevalence of 93.0% (66/71) was found in CN serum samples, which reacted with the VP1 L mimotope, whereas a prevalence of 88.7% (63/71) was reached with the VP1 M peptide (Table 1).

In serum samples from HS1 (*n* = 87), the prevalence of IgG antibodies against BKPyV VP1 L and VP M was 64.4% (56/87) and 74.7% (65/87), respectively (Table 1).

It is interesting to note that the prevalence of antibodies against BKPyV obtained with the two VP1 peptides L and M did not differ statistically within each group, which was 93.0% for peptide L vs. 88.7% for peptide M in CN group and 64.4% for peptide L vs. 74.7% for peptide M in HS1 group (Table 1). The prevalence detected in HS2 was 70.8% for peptide VP1 L and 71.5% for peptide VP1 M (Table 1), whereas it was ~73%, i.e., VP1 L (72.6%) and VP1 M (73.1%) in HS3 (Table 1).

In our study, sera were considered BKPyV VP1-positive when reacting with both mimotopes/peptides L and M.

When combining the data of BKPyV-positive sera, both for the VP1 L and VP1 M mimotopes, the overall prevalence was 87.3% (62/71) in CN and 62.1% (54/87) in HS1 (Table 1). The difference between the two prevalences, 87.3 vs. 62.1%, of the two cohorts is statistically significant (*p* < 0.0005).

A similar prevalence of BKPyV antibodies, 63.9 and 64.9%, was obtained in HS, when the number of control samples was double in HS2 (*n* = 144) and triple in HS3 (*n* = 208), respectively (Table 1).

BKPyV-positive sera tested by indirect E.L.I.S.A., diluted 1/20, had a general cut-off, by spectrophotometric reading, in the range of 0.17–0.19 OD. This value discriminates BKPyV-negative/-positive samples. Our indirect E.L.I.S.A. with BKPyV VP1 mimotopes was set up using rabbit BKPyV hyperimmune serum, which had an OD of up to 1.8, as a positive control. Conversely, the two negative controls, rabbits JCPyV, and SV40 hyperimmune sera, showed an OD of <0.1. A human peptide, represented by the neuropeptide S (hNPS), a.a sequence SFRNGVGTGMKKTSFQRAKS, which is BKPyV un-related, was used as a negative peptide in all indirect E.L.I.S.A. reactions. This negative control, which did not react with human sera under analysis, showed OD values in the 0.01–0.02 range, an OD reading which is expected for human sera tested BKPyV-negative (Pietrobon et al., 2017).

The two indirect ELISAs, with two distinct VP1 mimotopes gave overlapping results, thus confirming the higher prevalence of anti-BKPyV VP1 antibodies in human sera from patients affected by CN compared to controls (Table 1).

**Figure 1:**
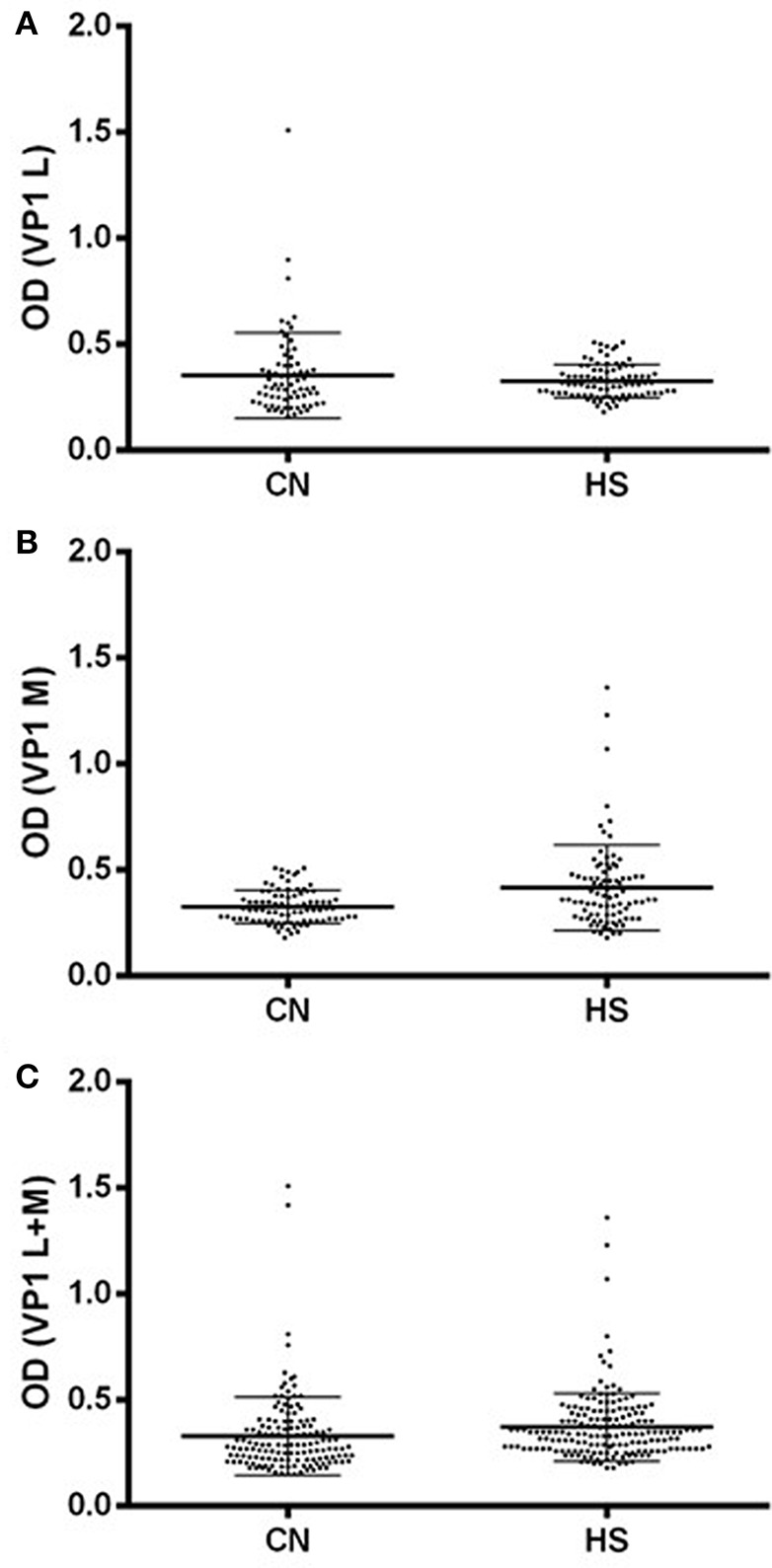
Serologic profile of serum antibody reactivity to BKPyV mimotopes VP1 L **(A)** and VP1 M **(B)** and VP1 L+M **(C)**. Immunologic data are from CN and HS serum samples. Results are presented as values of optical density (OD) readings at λ 405 nm of serum samples diluted at 1:20, detected by the indirect enzyme-linked immunosorbent assay. In scatter dot plotting, each plot represents the dispersion of OD values to a mean level indicated by the line inside the scatter with SEM for each group of patients/subjects analyzed. **(A)** The mean OD for VP1 L in CN and HS does not differ statistically, while **(B)** the mean OD of sera (VP1 M ± Std Error) in CN (0.326 ± 0.008) is higher than that detected in HS (0.417 ± 0.021). **(C)** The mean OD of sera (VP1 B+C ± Std Error) in CN (0.329 ± 0.015) is lower than that detected in HS (0.372 ± 0.012). Statistical analyses were performed using *t*-test.

Profiles of serum antibody reactivity to BKPyV mimotopes are presented in Figure 1. Immunological data are from samples from CN affected patients and HS. Results are presented as values of optical density (OD) readings at λ 405 nm, of serum samples diluted at 1:20, detected in indirect E.L.I.S.A. In scatter dot plotting, each plot represents the dispersion of OD values to a mean level indicated by the line inside the scatter with Standard Deviation (*SD*) for each group of subjects analyzed. The mean OD of sera (VP1 L ± Std Error) in CN (0.354 ± 0.024) does not differ from that of HS (0.326 ± 0.008), *p* > 0.005, whereas the mean OD of sera (VP1 M ± Std Error) in CN (0.326 ± 0.008) is higher than that detected in HS (0.417 ± 0.021), ^***^p < 0.001 (Figure 1C). The mean OD of sera (VP1 B+C ± Std Error) in CN (0.329 ± 0.015) is lower than that detected in HS (0.372 ± 0.012), ^*^p < 0.05. Statistical analyses were performed using *t*-test.

### BKPyV antibodies in human sera by haemagglutination inhibition (HAI) assay

The HAI method was also used to test for the presence of BKPyV antibodies in serum samples from CN and HS. To this purpose, sera from 71 CN and 87 HS, which had been serially diluted 1:16; 1:32; 1:64; 1:128, were analyzed by HAI. These serum dilutions were selected based on previous publications reporting that at higher dilutions BKPyV antibodies, which are present at lower titer, agglutinate human erythrocytes with a lower prevalence. Indeed, as shown in a recent publication by Pietrobon et al. (2017), the 1:128 dilution was chosen since other dilutions, such as 1:32 or 1:64, contain antibodies at higher concentrations, which may give rise to false positive reactions; on the other hand higher dilutions could carry antibodies at much lower concentrations giving false negative reactions (Mazzoni et al., 2012; Pietrobon et al., 2017). In our experience, higher dilution than 1:128 is not needed (Mazzoni et al., 2012; Pietrobon et al., 2017).

A prevalence of 91.5% (65/71) BKPyV-positive samples diluted at 1:128 was revealed in CN affected patients, while the prevalence was 64.4% (56/87) in the HS control group. In the HAI assay, the positive neutralization (immune serum) is present in those wells where human erythrocytes form a red botton/dot sedimented on the bottom. In these wells, human sera tested BKPyV-positive. The negative result (non-immune serum) is present in wells where the human erythrocytes are agglutinated by the BKPyV virion activity, thus forming a network of human red cells, which remain in suspension, showing a diffuse rose color in the solution. These sera are BKPyV-negative (data not shown).

As detected using the indirect E.L.I.S.A., the seroprevalence of BKPyV-positive samples among CN patients is higher than that determined in the control group of HS (91.5% vs. 64.4%; *p* < 0.001); the difference is statistically significant (Table 2).

**Table 2:** Prevalence of serum IgG antibodies against BKPyV analyzed by haemagglutination inhibition (HAI) assay.

**Human serum**	**Number of subjects/patients**	**Median age ± DS**	**Male (%)**	**CN/HS ratio**	**Number of positive samples (%)**		
				**1:16**	**1:32**	**1:64**	**1:128**
CN	71	67 ± 11	38	69 (97)	69 (97)	67 (94.3)	65 (91.5)^**^
HS	87	69 ± 13	41	85 (97.7)	85 (97.7)	84 (96.6)	56 (64.4)

Human sera were from patients affected by choroidal nevi (CN) and healthy subjects (HS). The prevalence of BKPyV antibodies in CN patients is statistically significant higher than that detected in HS (

** *p < 0.005)*.

*Statistical analysis was performed using the χ^2^ test*.

The concordance of BKPyV antibody prevalence determined using the two different assays is 97%.

## Discussion

In this investigation, the association between CN and BKPyV was analyzed using two methods, an indirect E.L.I.S.A. with synthetic peptides and HAI. Both methods are highly specific in detecting BKPyV antibodies in human sera. However, the indirect E.L.I.S.A. with mimotopes is simpler and faster, requiring less skill and tedious preparation than HAI.

To circumvent the problem related to the cross-reactivity among the three polyomaviruses BKPyV, JCPyV, and SV40 a more precise and sensitive immunologic method, such as the indirect E.L.I.S.A. employed herein (Pietrobon et al., 2017), was used to detect specific IgG antibodies against BKPyV.

Serum samples were considered BKPyV-positive only upon reaction with both peptides. Combining the data from the BKPyV-positive sera, the overall prevalence was 87.3% (62/71) in CN and 62.1 % (54/87) HS in a 1:1 ratio 63.9% (92/144) HS in a 1:2 ratio and 64.9% (135/208) a 1:3 ratio (Table 1). This difference is statistically significant (*p* < 0.0005).

Two indirect E.L.I.S.A.s, with two distinct VP1 mimotopes gave overlapping results, thus confirming the higher prevalence of anti-BKPyV VP1 antibodies in human sera from patients affected by choroidal nevi compared to controls (Table 1). No positive results were obtained with the human peptide hNPS used as a control.

Serologic profiles of serum antibody reactivity to BKPyV mimotopes show that seroprevalence is higher in CN compared to HS, but the antibody titre was lower in CN than in HS.

Human sera were also investigated by HAI. The seroprevalence of BKPyV-positive samples, diluted 1:128, was 91.5% (65/71) in CN affected patients, while in the control group, represented by HS, the prevalence was 64.4% (56/87). It is important to note that the seroprevalence of BKPyV antibodies in CN patients determined by HAI is higher than that detected in HS. The difference in the prevalence between the two cohorts, CN vs. HS (91.5% vs. 64.4%, ^**^P < 0.001), is statistically significant (Table 2).

The indirect ELISA was carried out with low dilution of serum/antibodies because in our ELISA the antigens are contained in two peptides with few a.a. and few epitopes, around ten each mimotope. HAI data were obtained at 128X, which is a high dilution of serum/antibodies. In this assay, antigens are represented by the complete virion, which may carry hundreds of epitopes per capsid. Thus, to detect specific antibody-antigen reactions in the two methods different dilutions are needed.

As mentioned above, this is the first investigation reporting data on the association between CN and BKPyV. At present, we cannot compare our study with the data of other investigation. Epidemiological data on BKPyV, published before, were obtained with immunological tests employing polyomavirus VP1 antigens that cross-react with other viruses of the family, i.e., JCPyV and SV40. Early prevalence data, due to the cors-reactiviy of the antigen employed, are probably over-estimated. In Western Countries BKPyV is wide-spread in different populations with a similar prevalence. BKPyV seems to be absent only in segregated populations living in Brazil, Paraguay, and Malaysia (Brown et al., 1975; Barbanti-Brodano et al., 1998).

In this study, indirect E.L.I.S.A.s, using BKPyV mimotopes from VP1 antigens, were employed for the detection of BKPyV antibodies in human sera from CN affected patients and HS. E.L.I.S.A. gave reliable results, which can be obtained on several samples over a short period of time with affordable costs. This E.L.I.S.A. may provide the scientific community with a standardized assay for the study of BKPyV infection in human populations and its association with other human diseases, including tumors.

To verify the data obtained by indirect ELISAs, another consolidated technique, the haemagglutination inhibition (HAI) assay was used. It turned out that data by HAI overlap the results obtained by the indirect E.L.I.S.A. with synthetic peptides.

In this context, it should be noted that in a previous investigation we did not find any association between CN and other small DNA tumor viruses, such as the polyomavirus SV40. Indeed, the prevalence of SV40 antibodies in serum samples from CN patients (17%) did not differ from that revealed in HS (15%; Bononi et al., 2014).

Our immunologic data indicate that the majority of CN (87%) is associated with BKPyV, a small DNA tumor virus.

Our study demonstrates with two independent and highly specific tests that the prevalence of BKPyV antibodies in sera of CN and HS differs significantly, although high in both groups. We think that these data will add new insight on the association between CN and BKPyV.

CN onset, like other human cancers, is due to specific gene mutations. Since BKPyV is oncogenic, clastogenic, mutagenic and a transforming viral agent, it may be a risk factor in CN onset/progression, alongside U.V. and other oncogenic agents. One may speculate that after infecting the host, BKPyV which remains latent lifelong may exert its transforming potential when the immune system is impaired. Indeed, the mean age of the CN cohort was 67 years old, which is compatible with a natural decline in the immune system, thus enabling BKPyV to reactivate and in some instances transform eye melanocytes. Indeed, the peculiar condition of the elderly host together with the impairment of the immune system, due to the age, do not allow all subjects to neutralize the potential oncogenic properties of BKPyV. A similar hypothesis has been proposed to explain the association between the prostate carcinoma and BKPyV (Keller E. X. et al., 2015; Keller X. E. et al., 2015; Tognon and Provenzano, 2015).

It should be noted that a higher prevalence of BKPyV antibodies in sera from CN affected patients compared to control is not proof of cause/effect in inducing CN by BKPyV.

In conclusion, this investigation reports on the association between choroidal nevi and BKPyV infection. Indeed, BKPyV antibodies were detected in CN serum samples with a higher prevalence than that revealed in HS sera. Our indirect ELISA with two BKPyV mimotopes appeared highly specific in detecting serum antibodies against BKPyV, without cross-reactivity with SV40 and JCPyV, which are closely related polyomaviruses. Our data are innovative and may represent a starting point for further investigations, together with additional factors, of the putative role of BKPyV in CN onset/progression. Indeed, the ability to detect only BKPyV without cross-reactivity with other two polyomaviruses, such as JCPyV and SV40, seems to be very valuable for additional studies in the field.

## Author contributions

MT, PP, and FM conceived and designed of the work; PP and SV performed the clinical characterization and provided samples; SP and FL performed the experiments; SP, IB, and EM analyzed and interpreted the data; SP, MT, and FM wrote the paper; EM, PP, and IB critically revised the manuscript. All authors read and approved the final manuscript.

### Conflict of interest statement

Data of this work were enclosed, in part, in the Italian patent application number I0167478/BRE-EC/rp, filed on August 9, 2016. The authors declare that the research was conducted in the absence of any commercial or financial relationships that could be construed as a potential conflict of interest.
